# Supplementary material for: Temporal nanofluid environments induce prebiotic condensation in water
Source: Commun Chem. 2023 Apr 14;6:69. doi: 10.1038/s42004-023-00872-y (PMC10104841; doi:10.1038/s42004-023-00872-y)
Supplement: Supplementary file 2 — Supplementary Information [file 42004_2023_872_MOESM2_ESM.pdf]

Supplementary Information

Temporal nanofluid environments induce prebiotic condensation in water

Andrea Greiner de Herrera [1,2,3], *Thomas Markert* [4], *Frank Trixler* [1,3,5*]

[1] Department of Earth and Environmental Sciences, Ludwig-Maximilians-Universität München, Theresienstraße 41, 80333 Munich, Germany.

[2] Center for Neuropathology and Prion Research (ZNP), Ludwig-Maximilians-Universität München, Feodor-Lynen-Str. 23, 81377 Munich, Germany.

[3] School of Education, Technical University of Munich and Deutsches Museum, Museumsinsel 1, 80538 Munich, Germany.

[4] Institute of Theoretical Chemistry, Ulm University, Albert-Einstein-Allee 11, 89081 Ulm, Germany.

[5] Center for NanoScience (CeNS), Ludwig-Maximilians-Universität München, Schellingtr. 4, 80799 Munich, Germany.

*corresponding author. e-mail address: trixler@lrz.uni-muenchen.de

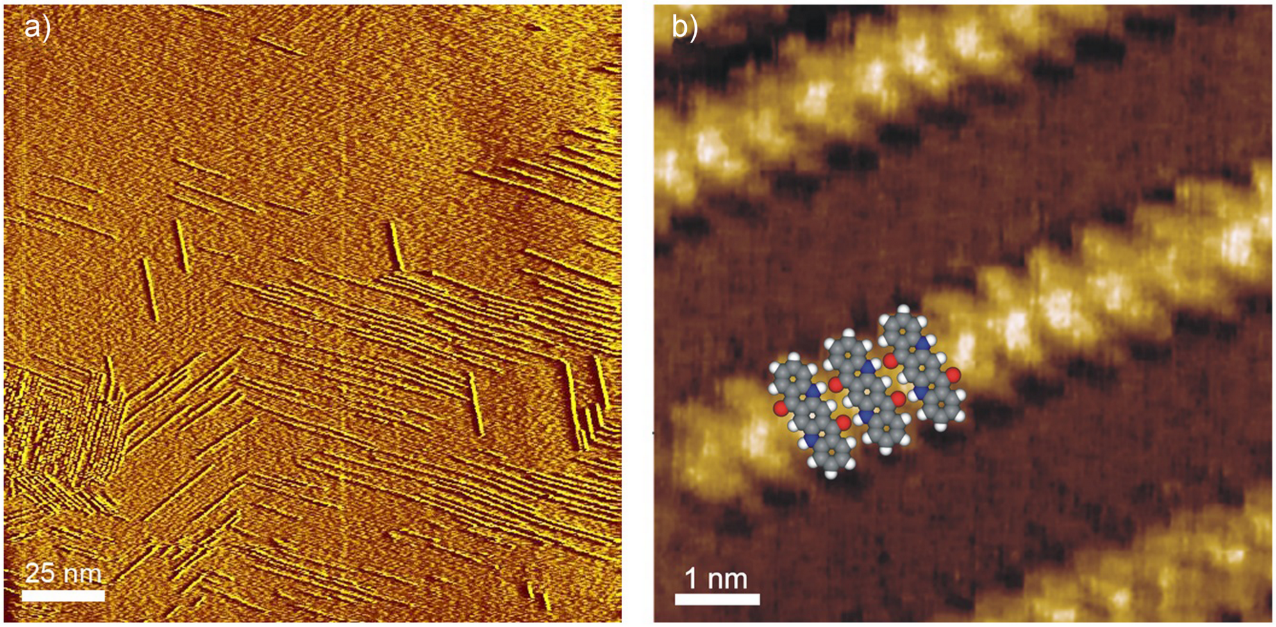

Supplementary Figure 1. **Scanning Tunneling Microscopy (STM) images of a graphite surface after organic solid/solid wetting deposition (OSWD) processing. a** Chain-like supramolecular adsorbate structures formed via OSWD after the graphite surface contacts suspensions of QAC particles. **b** Close-up view of a single supramolecular QAC adsorbate chain superimposed by a force field calculated assembly of three QAC molecules forming a linear chain via H-bonds.

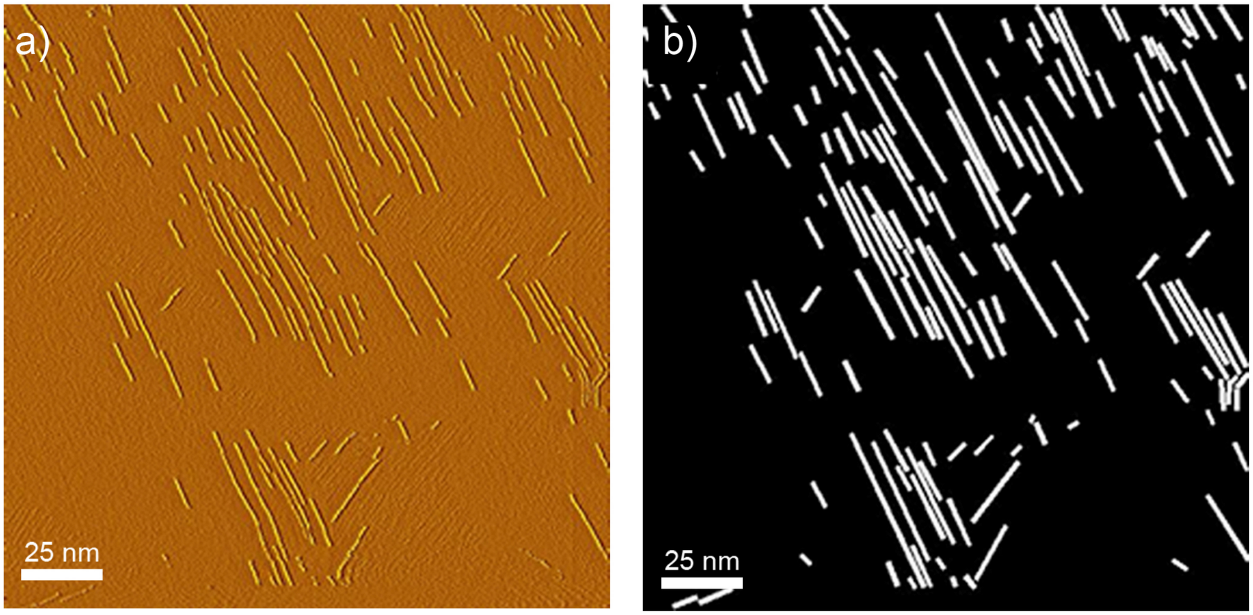

Supplementary Figure 2. **Example of a black/white conversion of STM images for coverage analysis. a** STM image of chain-like QAC assemblies on graphite. **b** Black/white conversion of the STM image: QAC chains were masked out (white) and the background was set to black. The percentage of the white proportion (the QAC coverage) was subsequently calculated as described in the methods section.

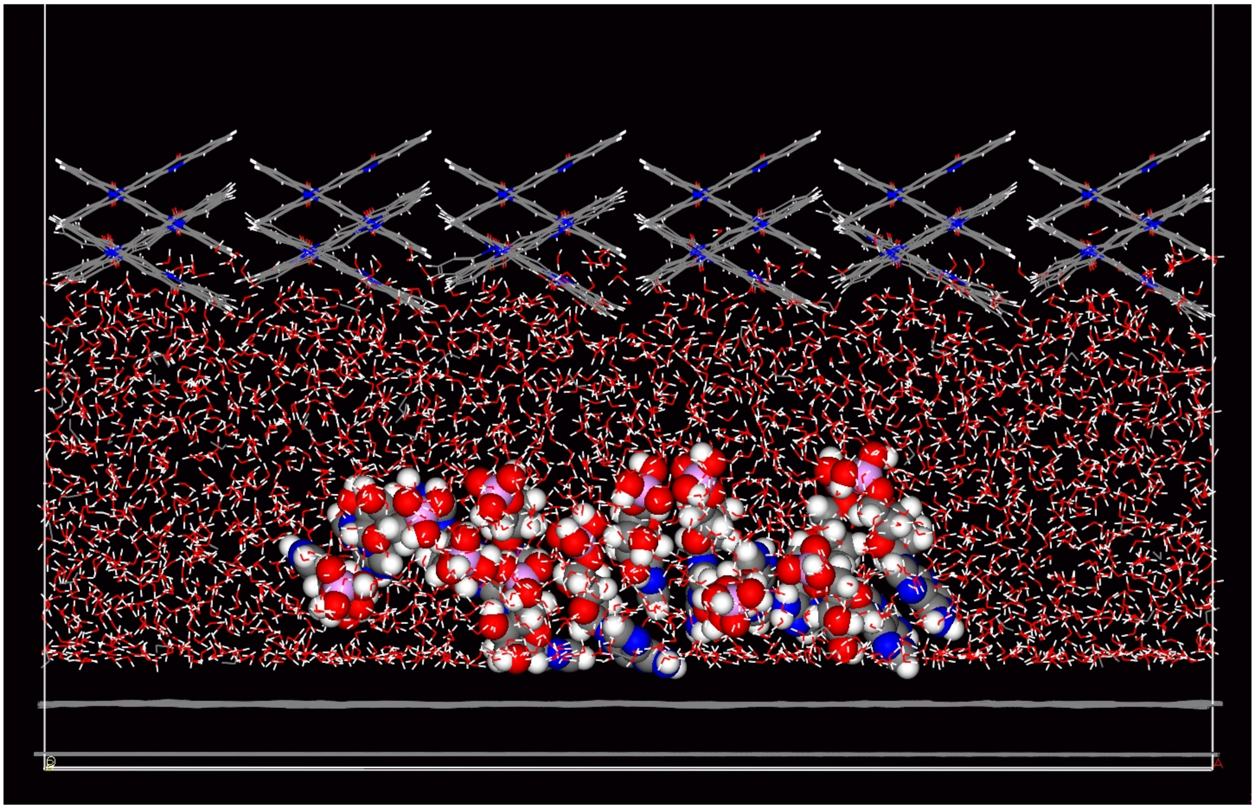

Supplementary Figure 3. **Dynamic force field calculation of an AMP stack within nanoconfined water**. The simulation was modeled with a reduced confinement gap size of 2.6 nm between a QAC crystal (top) and a graphene layer (bottom). Displayed is the condition of the stack after a simulated time span of 60 ps, revealing a stack in destabilized, non-ordered condition.

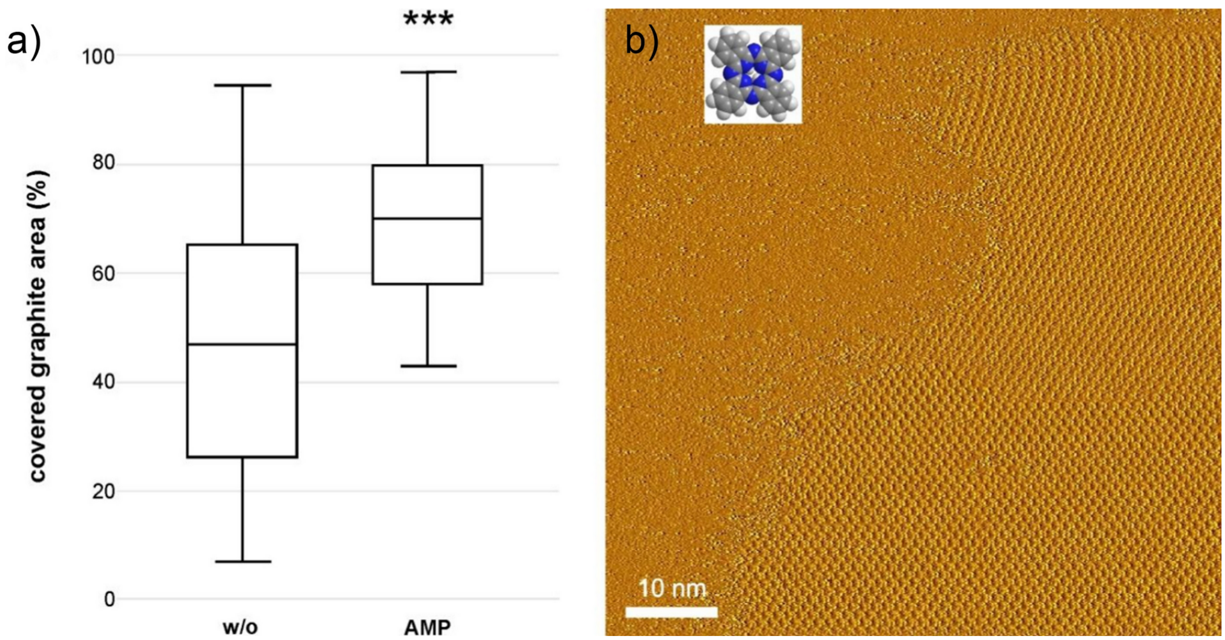

Supplementary Figure 4. OSWD induced coverage of graphite with monolayer of phthalocyanine (a widely used organic semiconductor[1] and model system for biochemical and prebiotic relevant porphyrines[2]). The monolayer that form after graphite has been brought into contact with a watery suspension of phthalocyanine particles. **a** Box plots based on surface coverage determinations via STM. The suspensions either contain dissolved AMP or no biomolecules (w/o). Testing: t-test with Welch´s correction; n=30 independent scans; ***: $p<0.001$. **b** Example STM image of a typical monolayer domain showing a densely packed supramolecular pattern that matches with the structure of phthalocyanine monolayer on graphite.[3] Inlay: molecular structure of a phthalocyanine molecule.

Supplementary References

(1) Zhang, Y., Cai, X., Bian, Y. & Jiang, J. Organic Semiconductors of Phthalocyanine Compounds for Field Effect Transistors (FETs) in *Functional Phthalocyanine Molecular Materials. Structure and Bonding* (ed. Jiang, J.) 275–321 (Springer, 2010). DOI: 10.1007/978-3-642-04752-7_9

(2) Pleyer, H. L., Strasdeit, H. & Fox, S. A possible prebiotic ancestry of porphyrin-type protein cofactors. *Orig. Life Evol. Biosph.* **48**, 347-371 (2018). DOI: 10.1007/s11084-018-9567-4

(3) Nilson, K. et al. Scanning tunneling microscopy study of metal-free phthalocyanine monolayer structures on graphite. *J. Chem. Phys.* **127**, 114702 (2007). DOI: 10.1063/1.2770732
