# Supplementary material for: Temporal nanofluid environments induce prebiotic condensation in water
Source: Commun Chem. 2023 Apr 14;6:69. doi: 10.1038/s42004-023-00872-y (PMC10104841; doi:10.1038/s42004-023-00872-y)
Supplement: Supplementary file 3 — Description of Additional Supplementary Files [file 42004_2023_872_MOESM3_ESM.pdf]

Description of Additional Supplementary Files

File name: Supplementary Movie 1.

Description: Simulation of an AMP stack within a confinement filled with water. Simulated via dynamic force field calculation is a supramolecular stack of 12 AMPs confined between a QAC crystal (top) and a graphene layer (bottom). A gap of 43 Å separates these confining surfaces and is filled with water. The simulation represents a time span of 50 ps, and the final state of the stack at the end of the simulation is depicted in Fig. 4a.

File name: Supplementary Movie 2

Description: Simulation of an AMP stack within a confinement with a water density reduced to approximately 60%. The dynamic force field calculation simulates a supramolecular stack of 12 AMPs assembled in nanoconfined water. The modeling parameters are identical to those in Supplementary Movie 1 with the exception of water density. The simulation represents a time span of 60 ps. The final configuration is shown in Fig. 4b.
